# Correction: Harmanci et al. Production of 3D Printed Bi-Layer and Tri-Layer Sandwich Scaffolds with Polycaprolactone and Poly (vinyl alcohol)-Metformin towards Diabetic Wound Healing. *Polymers* 2022, *14*, 5306

**DOI:** 10.3390/polym17212880

**Published:** 2025-10-29

**Authors:** Sena Harmanci, Abir Dutta, Sumeyye Cesur, Ali Sahin, Oguzhan Gunduz, Deepak M. Kalaskar, Cem Bulent Ustundag

**Affiliations:** 1 Center for Nanotechnology & Biomaterials Application and Research (NBUAM), Marmara University, Istanbul 34722, Turkey; 2 Department of Bioengineering, Faculty of Chemical and Metallurgical Engineering, Yildiz Technical University, Istanbul 34210, Turkey; 3 UCL Division of Surgery and Interventional Sciences, Royal Free Hospital Campus, London NW3 2PF, UK; 4 Department of Biochemistry, Faculty of Medicine, Marmara University, Istanbul 34722, Turkey; 5 Department of Metallurgical and Materials Engineering, Faculty of Technology, Marmara University, Istanbul 34722, Turkey; 6 Health Biotechnology Joint Research and Application Center of Excellence, Esenler, Istanbul 34220, Turkey

**Error in Figure**

In the original publication [1], there was a mistake in Figure 9 as published. Two images, labeled as “PCL/PVA-Met—7 days—10×” and “PCL/PVA-Met—7 days—20×,” were inadvertently taken from the 3-day group and mistakenly included in the 7-day panel during figure assembly. The corrected Figure 9 appears below. The authors state that the scientific conclusions are unaffected. This correction was approved by the Academic Editor. The original publication has also been updated.

## Figures and Tables

**Figure 9:**
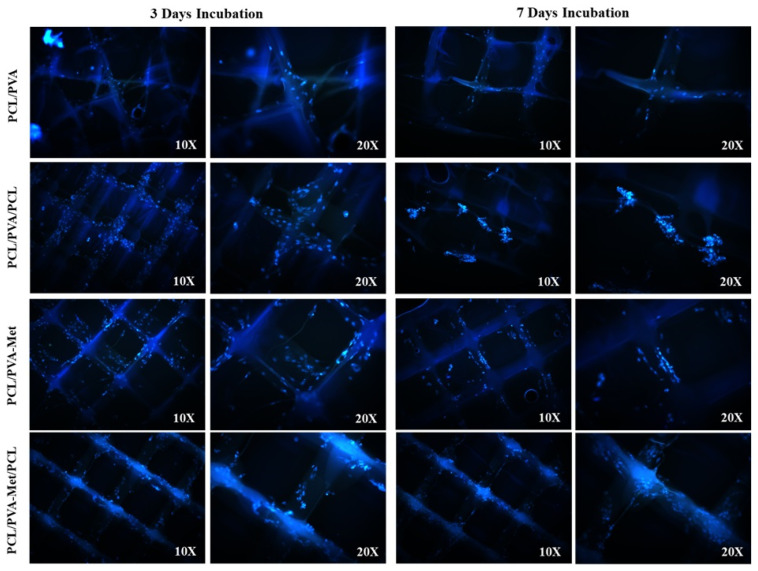
Fluorescent microscopy images of the DAPI stained fibroblast cells attached to the hybrid scaffolds, days 3 and 7 post-cell culture.

## References

[1] Harmanci S., Dutta A., Cesur S., Sahin A., Gunduz O., Kalaskar D.M., Ustundag C.B. (2022). Production of 3D Printed Bi-Layer and Tri-Layer Sandwich Scaffolds with Polycaprolactone and Poly (vinyl alcohol)-Metformin towards Diabetic Wound Healing. Polymers.

