# Supplementary material for: Production of 3D Printed Bi-Layer and Tri-Layer Sandwich Scaffolds with Polycaprolactone and Poly (vinyl alcohol)-Metformin towards Diabetic Wound Healing
Source: Polymers (Basel). 2022 Dec 5;14(23):5306. doi: 10.3390/polym14235306 (PMC9736052; doi:10.3390/polym14235306)
Supplement: Supplementary file 1 [file polymers-14-05306-s001.zip › polymers-1886984-supplementary.pdf]

Supplementary information

Figure S1. Standard curve for the Metformin

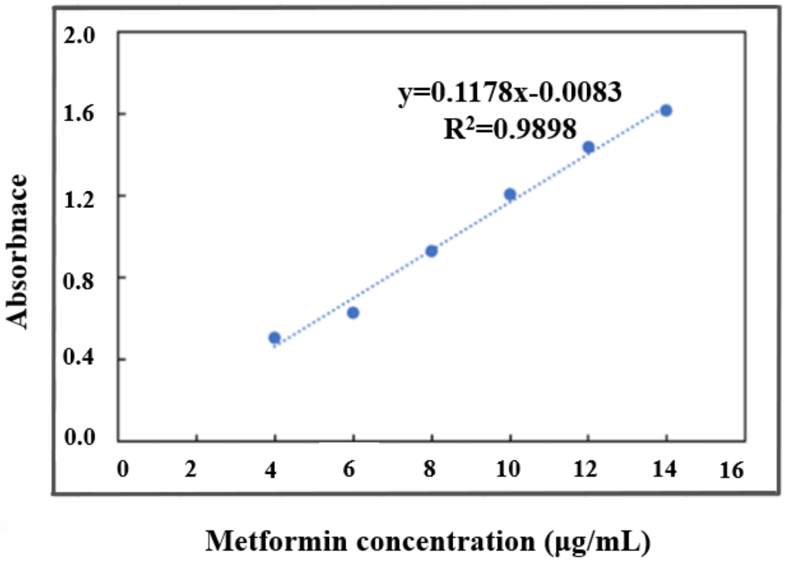

Figure S1. Standard curve for the Metformin.
